# Phase Composition of HiVac-VPE Lithium Niobate Optical Waveguides Identified by Spectroscopic Investigations

**DOI:** 10.3390/ma17102249

**Published:** 2024-05-10

**Authors:** Alicia Petronela Rambu, Sergey Kostritskii, Vyacheslav Fedorov, Oleg Sevostyanov, Irina Chirkova, Sorin Tascu

**Affiliations:** 1 Research Center on Advanced Materials and Technologies, Department of Exact and Natural Sciences, Institute of Interdisciplinary Research, “Alexandru Ioan Cuza” University of Iasi, Blvd. Carol I, No. 11, 700506 Iasi, Romania; alicia.rambu@uaic.ro; 2 RPC Optolink, Special’naya Territoriya Malogo Predtprenimatel’stva, Zelenograd, Sosnovaya al. 6A, 124489 Moscow, Russia; skostritskii@mail.ru (S.K.); fedorov@optolink.ru (V.F.); 3 Physics Department, Kemerovo State University, 650000 Kemerovo, Russia; olsevos@yahoo.com (O.S.); irina1989_2@mail.ru (I.C.)

**Keywords:** lithium niobate optical waveguides, Raman spectroscopy, IR reflection, IR absorption, UV-VIS absorption

## Abstract

High-index contrast lithium niobate waveguides, fabricated by the High Vacuum Vapor-phase Proton Exchange (HiVac-VPE) technique, are very promising for increasing both the optical nonlinear and electro-optical efficiencies of photonic integrated devices. So as to play this role effectively, it is mandatory to know the crystallographic phase composition of waveguides and the position of protonated layers for appropriate tailoring and optimization based on the intended applications. In addition, the estimation of structural disorder and electro-optical properties of the waveguides are also of high interest. Benefiting from Raman spectroscopy, IR reflection, IR absorption, and UV-VIS absorption, the H_x_Li_1−x_NbO_3_ phase compositions, as well as the structural disorder in waveguides, were determined. Based on experimental data on the shift of the fundamental absorption edge, we have quantitatively estimated the electro-optic coefficient r_13_ in as-exchanged waveguides. The electro-optical properties of the waveguides have been found to be depending on the phase composition. The obtained results allow for reconsidering the proton exchange fabricating process of photonic nonlinear devices and electro-optic modulators based on high-index contrast channel waveguides on the LiNbO_3_ platform.

## 1. Introduction

The fast growth of modern photonics applications, whether for the enhancement of nonlinear optical efficiency at the single photon level for quantum information applications, customization of photonics circuitry, and scalability of integrated photonics platforms, to name only these (a few), demand high-quality optical waveguides. This fact motivated attempts to adjust or identify new techniques in view of the fabrication of optical waveguides exhibiting a high-index contrast, low propagation losses, and preserved electro-optical and nonlinear optical properties.

Thanks to its excellent electro-optical and nonlinear optical properties, doubled by the relative easiness of producing waveguides, the congruent lithium niobate (CLN) crystal is a platform of choice for various integrated optics applications and photonic devices. Our recently reported developments on the fabrication of high-index contrast waveguides on CLN substrates by using High Vacuum Vapor-phase Proton Exchange (HiVac-VPE) are very promising. Indeed, the HiVac-VPE optical waveguides are characterized by very high-index contrast (∆*n_e_* > 0.1), preserved intrinsic nonlinearity of the crystal, and relatively low propagation losses [1]. To understand the origin of these very promising features, the HiVac-VPE waveguides deserve particular attention regarding the phase composition and position of protonated layers by comparing them with waveguides fabricated using proton exchange techniques that have been developed and well-known for a long time.

Various methods were used to study the H_x_Li_1−x_NbO_3_ phases in proton-exchanged LiNbO_3_ optical waveguides: X-ray diffraction, M-lines mode spectroscopy, secondary ion mass spectrometry, thermo-gravimetric analysis, differential scanning calorimetry, forward recoil spectrometry, Rutherford backscattering spectrometry, Raman spectroscopy, IR reflection spectroscopy, IR absorption spectroscopy, and UV-VIS absorption spectroscopy [1,2,3,4,5,6,7,8,9,10,11,12,13,14,15,16,17,18,19,20,21,22,23,24,25,26]. The existence of six to seven phases of H_x_Li_1−x_NbO_3_ (depending on the crystallographic orientation of the main surface of the crystal plate) has been established in waveguides fabricated using various proton exchange techniques, including Proton Exchange, Annealed Proton Exchange, Soft Proton Exchange, Vapor-phase Proton Exchange, and High Index Soft Proton Exchange [9,10,15,19,22,25,27]. The specific Raman and IR reflection/absorption spectra are observed for each phase [9,10,11,12,13,14,15,16,17,19,21,24]. Therefore, for identification of any H_x_Li_1−x_NbO_3_ phase in waveguides fabricated by other techniques, such as the case of our HiVac-VPE [1,2,3] or Reverse Proton Exchange [24], it is sufficient to use optical spectroscopy data. In addition, the micro-Raman technique gives the possibility for fast depth profiling of phase composition in any waveguides [17]. The UV-VIS absorption spectroscopy data allow for evaluation of the band gap and, thus, for estimation of the electro-optic coefficient in any waveguides.

In this article, based on optical spectroscopy methods such as Raman spectroscopy, IR reflection, IR absorption, and UV-VIS absorption, we investigate phase composition, depth profile, and electro-optic properties in waveguides fabricated by the HiVac-VPE technique [1,2,3]. As each H_x_Li_1−x_NbO_3_ phase has a specific spectrum, interesting and very particular information was identified on the waveguides subjected to the study. This approach yields interesting results that have not yet been reported, enabling us to understand the origin of the optical features of the HiVac-VPE waveguides. For any relatively new established technique such as HiVac-VPE, this type of study is absolutely necessary to understand the different properties and peculiarities of the new types of optical waveguides compared to those manufactured through already known and extensively used proton exchange techniques within the scientific community.

## 2. Samples Fabrication and Index Profiles Reconstruction

**Figure 1:**
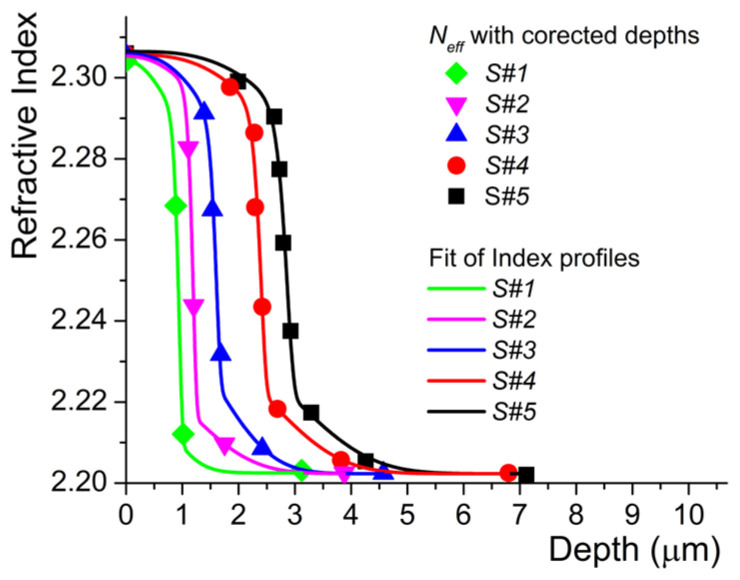
Index profiles of Z-cut HiVacPE waveguides fabricated for different exchange durations. The symbols represent the measured *N_eff_* of the propagating modes, except those on the ordinate that represent the IWKB corrected surface indices obtained by using the method detailed in references [1,3].

This study began by slicing six samples from a Z-cut CLN optical-grade wafer (Gooch & Housego, Crystal Technology, Palo Alto, California, United States). The samples are *w* = 8 mm in width and *l* = 15 mm long and were labeled S#0, S#1, S#2, and so on until S#5, respectively. The S#0 sample will be kept virgin and will be used as a reference to compare the results obtained on the protonated samples. The other five samples were protonated to create a planar waveguide on the Z^+^ top surface by using the HiVac-VPE technique, a process very well described in our previous work [1]. The waveguides were fabricated by exposing the samples to acid vapor for different exchange durations *t*(h) at an exchange temperature of *T_exch_ =* 350 °C. Each sample is introduced in a hermetically sealed hourglass-shaped glass tube. Prior to being sealed, the bottom part of the glass tube was filled with 16 g of Benzoic Acid (BA) (Sigma-Aldrich, St. Louis, MO, USA) powder as a vapor source. The purity of the powder is 99%. The sample is placed in the top part of the tube, and then, by using a turbo pumping system HiCube 80 Eco (Pfeiffer, Asslar, Germany), the tube is pumped down to a pressure as low as *p* = 4 ± 0.1 × 10^−5^ mbar. After the glass tube is sealed, it is placed into a homemade metallic tube container, which avoids thermal shocks and ensures uniform heating and easy and safe manipulation. The metallic tube is placed vertically in an oven (Lenton WHT5/60, Thermal Design, Hope Valley, UK) preheated at 350 °C, which is the exchange temperature. The exchange duration *t*(h) is incremented by 1 h, starting from *t* = 1 h for sample S#1 up to *t* = 5 h for sample S#5, respectively. It is important to note the fact that this technique is manifold because, besides both high-index contrast and preserved nonlinearity of the CLN optical waveguides, the HiVac-VPE process assures very high stability and reproducibility of optical waveguide features compared to current techniques [1,2,3]. The index contrast and the shape of index profiles are of particular importance, bringing the first information about the waveguides after their fabrication. In our study, for a given waveguide, the effective indexes *N_eff_* of the guided modes have been assessed by M-lines measurements using a two-prism setup at 632.8 nm laser wavelength (Thorlabs HTPS-EC-1 He-Ne Tunable Laser, Newton, NJ, USA). The precision of effective indexes *N_eff_* measurements is ± 2.5 × 10^−4^. Starting from *N_eff_* values, the reconstruction of the index profile of each planar waveguide was performed using the Inverse Wentzel–Kramers–Brillouin (IWKB) numerical method as described in [1]. The value of the index contrast exhibited by each waveguide is expressed as ∆*n_e_* and is calculated as the difference between the surface index given by IWKB and the extraordinary index value of the substrate (*n_e_* = 2.2028 in our case for Gooch & Housego virgin substrate at room temperature). The reconstruction of the index profile for such HiVac-VPE waveguides is presented in Figure 1, where symbols represent IWKB corrected surface indices on the ordinate and measure *N_eff_* of the propagating modes for the others, respectively. The solid line is the fit obtained by the sum of two generalized exponential functions, described by Equation (1):

(1) $$n\left(d\right)=n_{e}+A_{1}\exp\left(-{\left(\frac{d}{w_{1}}\right)}^{a_{1}}\right)+A_{2}\exp\left(-{\left(\frac{d}{w_{2}}\right)}^{a_{2}}\right)$$

where *A*_1_, *A*_2_, *w*_1_, *w*_2_, *a*_1_, and *a*_2_ are adjustable parameters and take different values depending on exchange time, and *d* is the depth of the waveguide. The method is detailed in our previous works [1,3]. Table 1 summarizes the index contrast values ∆*n_e_* @ *λ* = 632.8 nm of the samples under investigation.

As can be seen, the samples exhibit almost the same index contrast due to the same acidity, but because different exchange durations were used, the depth of the waveguide is the only difference between such fabricated waveguides.

**Figure 2:**
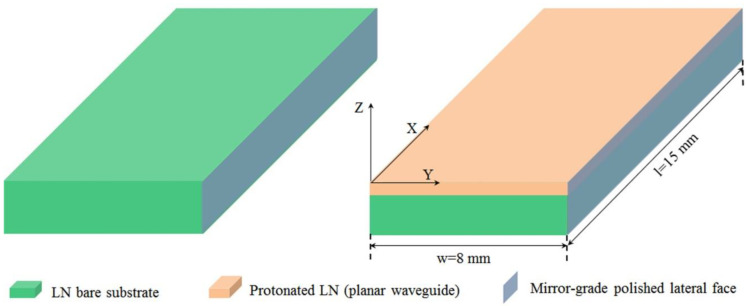
Geometry of HiVac-VPE waveguides as prepared for spectroscopic investigation. Protonated and bare CLN substrate was mirror-grade polished on a lateral face.

After this characterization, in view of spectroscopic investigations, all six samples, the virgin substrate, and proton-exchanged waveguides, were mirror-grade polished on a lateral face, as sketched in Figure 2.

At this stage of our study, the six samples were investigated, respectively, by Raman spectroscopy, IR reflection, IR absorption, and UV-VIS absorption, thus making it possible to determine the phase composition of protonated layers. Once the characterization of all planar waveguides was completed, we decided to anneal sample S#1 with the goal of obtaining a waveguide exhibiting α-phase only in the protonated layer. The other samples require a much longer annealing time to achieve this goal, and the resulting waveguides will be very deep (useless in view of practical applications). Using the annealing process, the S#1 sample became a new one and was labeled S#1a. The results on Raman spectroscopy, IR reflection, IR absorption, and UV-VIS absorption obtained on the S#1a sample will be presented in the next section, together with the results obtained on the other samples. This will facilitate comparison and discussion of the obtained results.

**Figure 3:**
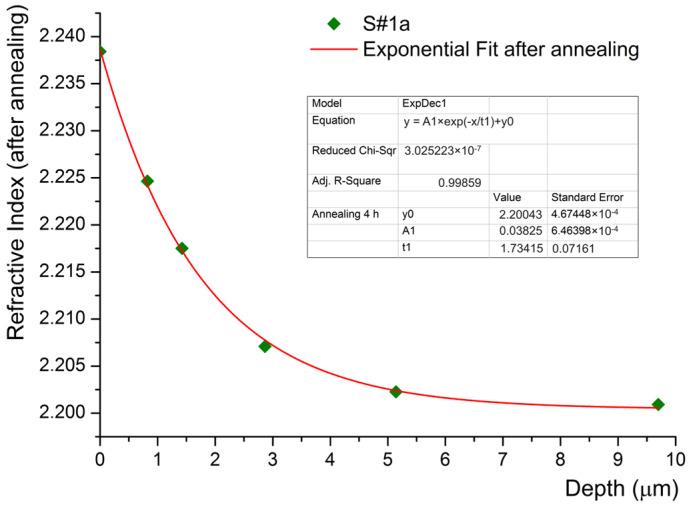
Index profiles of S#1a sample annealed at *T_anneal_ =* 360 °C for 4 h. The symbols represent the measured *N_eff,anneal_* of the propagating modes, except the ones on the ordinate that represent IWKB-calculated surface indices.

The changes induced by an annealing process on both the shape of the index profile and the index contrast value of sample S#1 were investigated. The annealing was carried out for 4 h in normal atmospheric conditions by introducing the samples in a preheated oven at *T_anneal_ =* 360 °C. In order to avoid thermal shocks and for easier and safer manipulation, the sample was placed in a glass tube open at both ends. After the annealing process, the sample was investigated following the same experimental protocol, i.e., reconstruction of index profiles and assessment of index contrast by using M-lines measurements and the IWKB numerical method, respectively. The first observation is that the number of modes and the shapes of index profiles completely change after annealing, as depicted in Figure 3. At the same time, the depth of the waveguides increases, a fact as natural as possible due to the further diffusion of protons into the crystal.

As anticipated, we notice a decrease in the index contrast from ∆*n_e_* = 0.1014 to ∆*n_e_* = 0.0332 after 4 h annealing. Therefore, the plots indicate that the shape of the index profile decreases exponentially. It seems that this aspect is another peculiarity of HiVac-VPE waveguides if compared with the literature devoted to Annealing Proton Exchange (APE) waveguides. For the APE waveguides, a generalized Gaussian function was often found to provide the best approximation for the shape of the index profile after annealing [4,5]. Our findings are very important in view of already-existing results reported by literature concerning the effect of the annealing and annealing time on the shape of the index profile of the waveguides fabricated by the APE technique. We assume that it can be explained by the fact that in the case of the APE technique, after proton exchange and before annealing, the waveguides exhibit a step-like index profile. This shape of the index profile is the initial condition found in any modeling and experimental study of annealing in the case of the APE technique, which is not the case in our investigation [4,5,6,7,8]. Our initial conditions are quite different: the index profile is expressed as the sum of two generalized exponential functions, as presented in Figure 1, and in agreement with our previous work [1].

To determine the phase composition of the waveguides, we took into account the data provided by Raman spectroscopy, knowing that each H_x_Li_1−x_NbO_3_ phase has a specific spectrum [9,10,11,12]. Proton exchange influences both vibration modes in Raman spectra, causing intensity reduction of some of the Raman lines, the appearance of new ones, and the appearance of disorder-induced broadening of some Raman bands [9,10,11,12,13,14,15,16,17,18]. The Raman spectra were measured with a LabRAM HR800 spectrometer (Horiba Scientific, Jobin Yvon S.A.S. Villeneuve d’Ascq, France) equipped with a confocal microscope. In this device, a linearly polarized laser beam was focused, using a ×100 microscope objective, on the surface of the investigated sample. Raman spectra were measured with a He-Ne laser (*λ* = 632.8 nm, *P* = 10 mW), confocal hole with 50 μm diameter (spatial resolution: lateral ~1.0 μm and vertical (in depth) ~1.5 μm for the photometered area) and a grating with 1800 tr/mm (spectral resolution = 0.8 cm^−1^). By Raman spectroscopy, the depth profile *d* (μm) of the proton-exchanged layer was obtained by moving the focused laser beam starting from the surface towards the substrate by steps in the range of 0.1–0.5 μm. The laser beam was directed onto the polished side surface (Y-cut, as shown in Figure 2). Depth profiling with a high spatial resolution (≤0.3 µm) was achieved by analyzing Raman spectra through this polished side surface. It is important to note that the side surface was polished after HiVac-VPE processing. The confocal microscope integrated into the Raman spectrometer, along with the precision and repeatability of the computer-controlled motorized stage, enabled this high spatial resolution. The effectiveness of this approach for depth profiling was demonstrated in reference [17].

Measurement of both IR reflection and IR absorption spectra was performed using a Bruker Vertex 80V spectrophotometer (Bruker Optics GmbH, Ettlingen, Germany) with standard attachments. The transmission spectra in the visible and near UV ranges were obtained using a Shimadzu UV-3101PC spectrophotometer (Shimadzu Corporation, Shimadzu Europe, Duisburg, Germany).

## 3. Results and Discussion

### 3.1. Raman Spectroscopy

**Figure 4:**
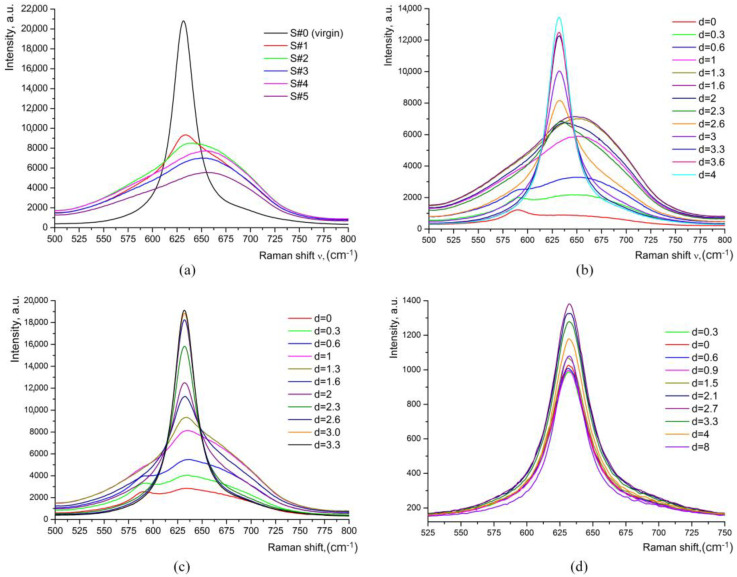
Raman spectra of HiVac-VPE waveguides measured within a range 500–800 cm^−1^: (**a**) Spectra of all samples measured at *d* = 1.3 μm; (**b**) Spectra of the S#3 sample measured at different values of *d*; (**c**) Spectra of the S#1 sample measured at different values of *d*; (**d**) Spectra of the annealed S#1a sample measured at different values of *d*. In this case, the laser power was much smaller in comparison with the measurements shown in (**a**–**c**). In addition, the integration time is decreased 5 times. The spectra were obtained step by step while the laser beam was crossing the polished edge (Y-cut, see Figure 2) of a sample from the surface towards the substrate. Polarization geometry was Y(ZZ)Y.

The HiVac-VPE process induces the extra band (with intensity *I*_x_) at 650–670 cm^−1^ and no band at 690 cm^−1^ in Raman spectra measured in the Y(ZZ)Y, Y(XX)Y, and Z(XX)Z geometries, as can be seen in Figure 4. These geometries were chosen to facilitate the study of all phonons with various symmetries (A1(TO), A1(LO), E(TO), and E(LO), respectively). Note that some spectra were recorded using a much smaller laser power by using a neutral density filter *D* = 1 instead of *D* = 0. This does not affect the shape and quality of the spectra but can reveal whether there is an influence of photorefractive effects on Raman spectra. In our experimental investigation, we did not identify any influence of photorefractive effects on Raman spectra.

**Figure 5:**
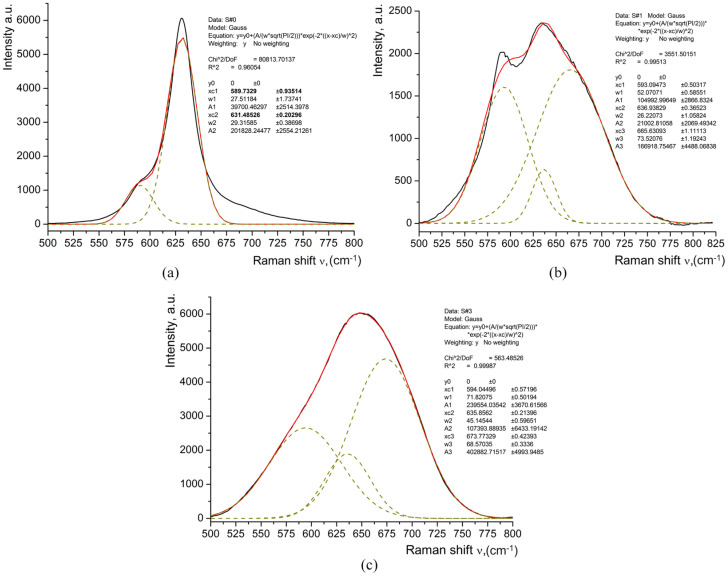
Decomposition of the Raman band observed at 500–800 cm^−1^ in some studied samples: (**a**) S#0 for *d* = 0 μm, elementary bands are 589.7 and 631.5 cm^−1^; (**b**) S#1 for *d* = 0 μm, elementary bands are 593.1, 636.9, and 665.3 cm^−1^; (**c**) S#3 for *d* = 1.6 μm, elementary bands are 594.1, 635.9, and 673.8 cm^−1^.

For some samples, the decomposition of the Raman band was observed in the range of 500–800 cm^−1^, as presented in Figure 5.

It is worth noting that the “paraelectric band” at 690 cm^−1^ is a specific feature of the *β_i_*-phases [9,10,12,13,14,15,16]. For example, in Proton Exchange (PE) waveguides with *β_i_*-phases, the double peak (630 and 690 cm^−1^) is observed [9,12,13,14,17]. In APE waveguides after short-time annealing, the broad band centered with a maximum at 652–656 cm^−1^ and a broad shoulder at ~660–676 cm^−1^ is observed [9,10,12,13,14,17], similar to spectra of the investigated HiVac-VPE samples. These APE waveguides contain the *κ*_2_-phase in accordance with the previous findings [9,10,12,13].

**Figure 6:**
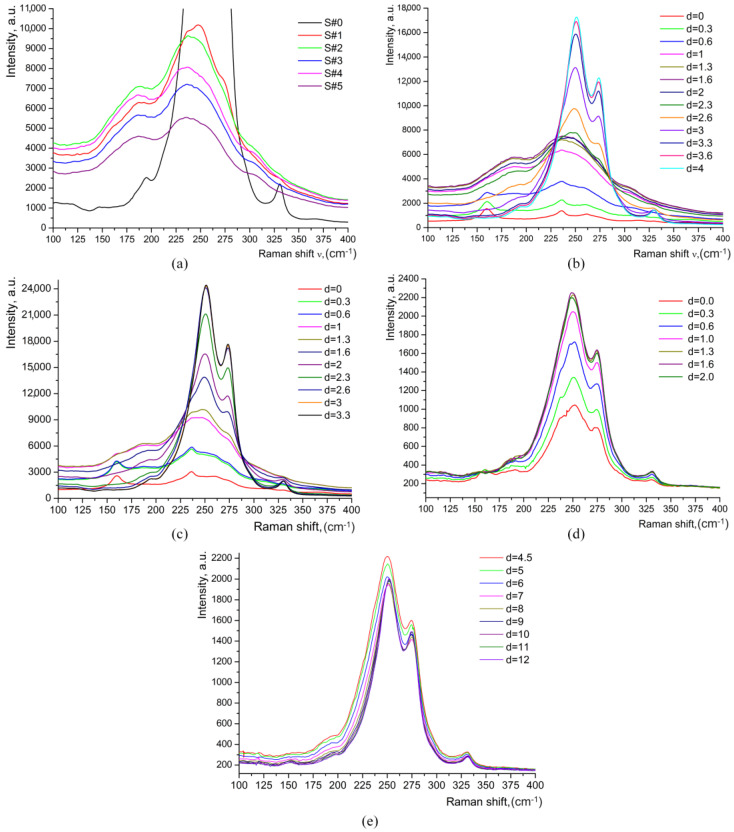
Raman spectra of the HiVac-VPE waveguides measured within the range of 100–400 cm^−1^: (**a**) Spectra of all samples measured at *d* = 1.3 μm; (**b**) Spectra of the S#3 measured at different values of *d*; (**c**) Spectra of the S#1 sample measured at different values of *d*; (**d**) Spectra of the S#1a measured for a near-surface part of the waveguide at different values of *d*; (**e**) Spectra of the S#1a measured for a depth part of waveguide at different values of *d*. Note that for the measurements shown in (**d**,**e**), the laser power was much lower than the measurements shown in (**a**–**c**). The polarization geometry was Y(ZZ)Y.

In addition, the other “paraelectric bands” at about 136 and 214 cm^−1^, which are specifically linked to *βi* phases [13,14,17], are not observed in the Raman spectra of the HiVac-VPE waveguides, as can be seen in Figure 6.

Raman spectra measured in all studied geometries demonstrate the layered multiphase structure of the HiVac-VPE waveguides: the *κ*_2_-phase in a top sublayer (with the anomalous thin near-surface part), the *α*-phase in a depth-thick sublayer, and some phase in a transient thin sublayer placed between the *κ*_2_-phase sublayer and *α*-phase sublayer, respectively.

We conclude that a HiVac-VPE waveguiding layer contains a sublayer having different values of *x* (decreasing towards the substrate), i.e., presenting a different phase in addition to the *k*_2_ and *α* phases. It should be the *k*_1_-phase that, according to references [13,14], is almost indistinguishable spectroscopically from *α*-phase since positions of the Raman peaks are just the same, i.e., no qualitative difference between the spectra of *α*- and *k*_1_-phases. However, our study [9,15] has shown that there is a marked quantitative difference between these spectra. This difference is related to the intensity of the band at 645–656 cm^−1^. Thus, in our opinion, the Raman spectra observed for the HiVac-VPE waveguides are typical, depending on depth, for the *κ*_2_, *κ*_1_, and *α* phases only.

In order to compare our data with that reported in the literature [13], we denote the Raman intensity measured at 631–635 cm^−1^ as *I*_630_ and, respectively, as *I*_690_, the Raman intensity measured at 690 cm^−1^, independently of the result of decomposition of Raman spectra at 500–800 cm^−1^. The relation between Raman data (Figure 4 and Figure 5) and phase composition is given in Table 2.

Although not very precisely, this dependence repeats the form and width of the optical profile, i.e., the Raman depth profiling of the samples (see Table 3) agrees well with the data extracted from Figure 1 and from reference [1] on refractive index profiles obtained with the M-line method.

**Figure 7:**
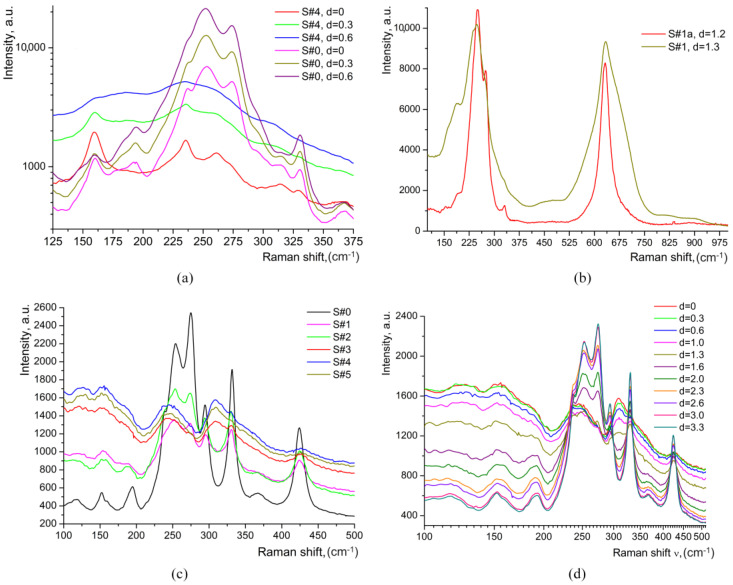
Low-frequency Raman scattering (LFRS) spectra measured in different samples: (**a**) for a very surface layer of the virgin S#0 sample and S#4 waveguide; (**b**) S#1 and S#1a waveguides, spectra measured at *d* = 1.2–1.3 μm. Spectra of S#1a waveguide were normalized to the spectra S#1 at 1000 cm^−1^ (polarization geometry was Y(ZZ)Y for the spectra (**a**,**b**)); (**c**) Spectra of all the samples measured with polarization geometry Y(XX)Y; (**d**) Spectra of S#4 waveguide measured at different values of *d* with polarization geometry Y(XX)Y.

At this moment of our investigation, an extra study was performed in order to decide which of the two hypotheses suggested in reference [14] is valid: (i) the existence of a thin, very surface layer with a different structure, or (ii) an experimental artifact of Raman spectroscopy caused by the probing of the air–layer interface. Through low-frequency Raman scattering (LFRS) [28,29,30], we obtained evidence of significant structural disorder induced by proton exchange in all HiVac-VPE waveguides. The tail of the broad LFRS for all the waveguides strongly increases with increasing of HiVac-VPE exchange time, as shown in Figure 6a–c and Figure 7 below.

**Figure 8:**
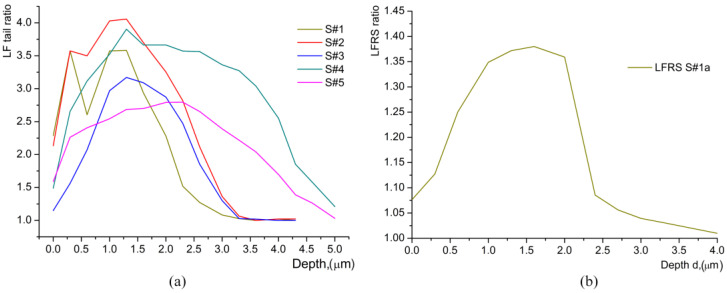
Low-frequency Raman scattering (LFRS) ratio versus *d*: (**a**) For all as-exchanged waveguides; (**b**) For S#1a (annealed S#1 waveguide). LFRS were measured in Y(ZZ)Y geometry. LFRS ratio is *I*_x_/(*I*_S#0_ − *I*_bg_) at ν = 100 cm^−1^ for various *d*. *I*_bg_ denotes the background intensity for the Raman spectra of the S#0 sample.

The intensity of LFRS at 100 cm^−1^ in the HiVac-VPE waveguides depends non-monotonously on depth *d*, as can be seen in Figure 8.

The strong and broad tail (extended up to 400–500 cm^−1^) of LFRS in the HiVac-VPE samples is similar to the LFRS in the strongly disordered media (e.g., glass) with order parameter within several tens of nanometers [28,29]. LFRS is also observed in Y(XX)Y geometry, as shown in Figure 7c,d.

After annealing of S#1 for 4 h, the bands associated with HiVac-VPE expand deeper into the S#1a sample (see Figure 4 and Figure 6). The Raman spectra are typical for the *α*-phase, as the extra bands specific for any *κ_i_* and *β_i_* phases are not observed (Figure 4, Figure 5, and Figure 6, respectively). The depth of a layer with the changed Raman spectra is increased up to *d_α_* ≈ 9 μm (see Figure 4 and Figure 5) after annealing in comparison with *d_α_* ≈ 3.3 μm (Figure 4 and Figure 5) for the sample before annealing. LFRS is decreased after annealing, as can be seen in Figure 4, Figure 5 and Figure 8.

The existence of the E(TO)-bands (152, 236, 265, 325, and 582 cm^−1^) in the very surface spectra, as can be observed in Figure 3, Figure 4, Figure 5, and Figure 6, respectively, is related by us to an experimental artifact caused by the probing of the air–sample interface, according to the finding reported in reference [14]. At the same time, the anomalous attenuation of Raman intensities for A_1_(TO)-bands 253 and 630–637 cm^−1^ (comparative to S#0 sample) and for the extra HiVac-VPE-induced bands demonstrates the existence of a very surface thin layer (≤0.6 μm) with either a different structure [14], or with a large intrinsic stress [10].

### 3.2. IR Reflection Spectroscopy

**Figure 9:**
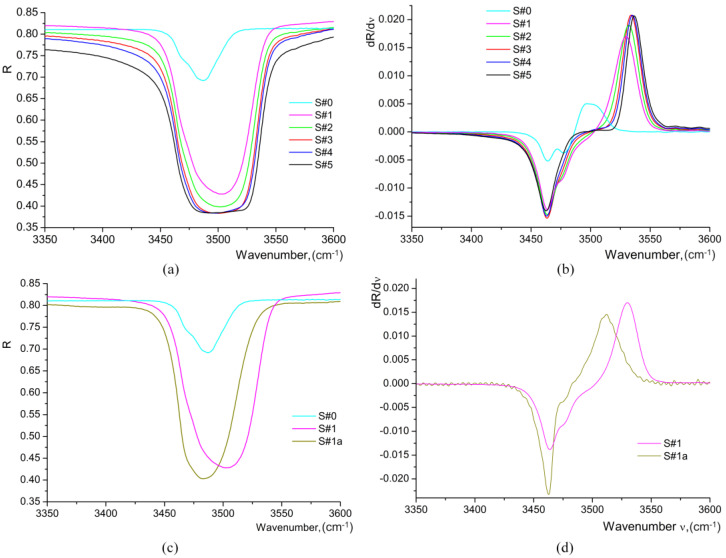
IR reflection spectra measured within the stretching vibration of OH-band at θ = 80° from a main Z-cut surface of a waveguide: (**a**) IR reflection spectra for all samples; (**b**) 1st derivative of IR reflection spectra of all samples; (**c**) IR reflection spectra for S#0, S#1, and S#1a; (**d**) 1st derivative of IR reflection spectra of S#1 and S#1a.

It was established [9,10,11,19,20,21,31,32,33] that the hydrogen in LiNbO_3_ forms an OH^−^ complex with the oxygen of the crystal lattice. The presence of this complex, confirmed by the characteristic IR-active stretching vibration band at about 3500 cm^−1^, was clearly identified in HiVac-VPE waveguides, as can be seen in Figure 9.

**Figure 10:**
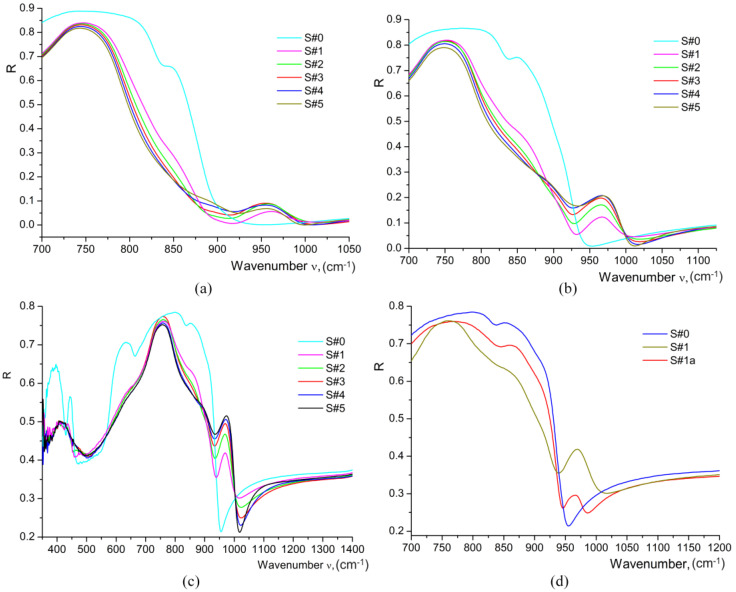
IR reflection spectra of all HiVac-VPE waveguides measured at different *θ* within the lattice vibration range: (**a**) *θ* = 20°; (**b**) *θ* = 60°; (**c**) *θ* = 80°; (**d**) IR reflection spectra of S#1 sample comparative to annealed S#1a and virgin S#0 samples measured at *θ* = 80°.

It is worth noting that the frequency and shape of this band depend on the phase composition of the waveguide. Thus, measurement of the OH band in the IR reflection spectra can be used for evaluation of the phase composition [9,19,21]. It is worth noting that the comparison of IR reflection spectra measured at different angles (20°, 60°, and 80°) demonstrates the multilayered structure of waveguides, with the *α*-phase in the deeper part of the protonated layer. Since the photo-metered depth *d_IR_* (penetration depth of IR beam) depends on the angle of incidence *θ*, at smaller angles (closer to the normal incidence), deeper penetration takes place, and the spectra measurements are affected by the presence of the various phases forming the waveguiding layer. It was established [9,15,19,21] that at *θ* ≥ 70° the spectrum of the near-surface sublayer is separated from those of deeper situated sublayers in the multiphase waveguides. Thus, the cases when *d_IR_* is much smaller than the thickness of the HiVac-VPE protonated layer are shown in Figure 10c,d. Moreover, it is seen that the spectra measured at *θ* = 20° are close to the shape of the spectrum of the virgin sample (S#0), suggesting that the contribution of the *α*-phase is more significant than those at larger incidence angles.

For determination of the phase composition of waveguides, we also used the IR reflection spectroscopy data in the region of the lattice vibrations of the crystal (100–1100 cm^−1^), the results being represented in Figure 10.

A comparative analysis shows that there are significant differences between the basic parameters of these spectra for different H_x_Li_1−x_NbO_3_ phases [9,11,19,21]. Thus, the proton exchange leads to the appearance of new phonons in the lattice vibration spectrum. This indicates both a change in the chemical bond parameters and the appearance of new chemical bonds. Thus, in the IR reflectance spectra of the studied HiVac-VPE waveguides, we observe additional bands in the range of 960–1050 cm^−1^ [9,15,19,21], which are absent in the spectrum of the virgin niobate crystal as depicted in Figure 9. The frequencies of these additional bands coincide with the characteristic frequencies of the librational vibrations [18] of the OH groups detected by Raman spectroscopy methods in rutile crystals [34]. This fact, together with the presence of a characteristic dependence of their frequencies on the proton concentration, allows us to assign all components of the additional bands to librational vibrations of OH groups at different lattice positions and ordered by means of weak hydrogen bonds of different lengths. These new bands observed at 970 and 965 cm^−1^ in the IR reflection spectra for all HiVac-VPE waveguides studied are presented in Figure 10. These OH-libration bands were assigned to the *κ*_2_-phase and to both the *κ*_1_-phase and *α*-phase because the *κ*_1_-phase and *α*-phase have the same frequency for this band in Raman spectra, respectively [13,14,19,21]. The bands at 975 cm^−1^ and 980 cm^−1^ attributed to *β*_1_, *β*_2_, and *β*_3_ phases [21] are not observed in the IR reflection spectra of HiVac-VPE waveguides. The new bands at 800–900 cm^−1^, which are specific for *β_i_* phases, are also not observed. Therefore, no *β_i_* phase exists in all investigated HiVac-VPE waveguides, according to the existing literature [9,11,21].

**Figure 11:**
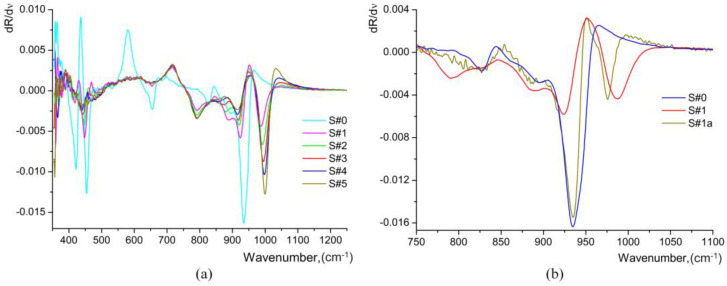
The 1st derivative of IR reflection spectra measured at *θ* = 80°: (**a**) for all HiVac-VPE samples (corresponding to the spectra shown in Figure 10c); (**b**) for the S#1 sample comparative to annealed S#1a and virgin S#0 (corresponding to the spectra shown in Figure 10d).

The frequencies of optical phonons can be determined from the position of the inflection points on the spectral dependence of the reflection coefficient *R*. For accurate determination of the frequencies of the inflection points, it is convenient to use the spectral dependence of the first derivative of the reflection coefficient with respect to the wavenumber *dR*/*d*ν [9,15]. In this case, the maxima on the *dR*/*d*ν curve correspond to the frequencies of *TO* phonons, while the minima correspond to the frequencies of *LO* phonons, as depicted in Figure 11.

The first derivative spectra for the OH-libration band at 950–970 cm^−1^ are similar for all the HiVac-VPE samples and show a slight gradual change (Figure 11), in contrast to PE/APE and SPE samples reported by the literature [9,15,21].

### 3.3. IR Absorption Spectroscopy

**Figure 12:**
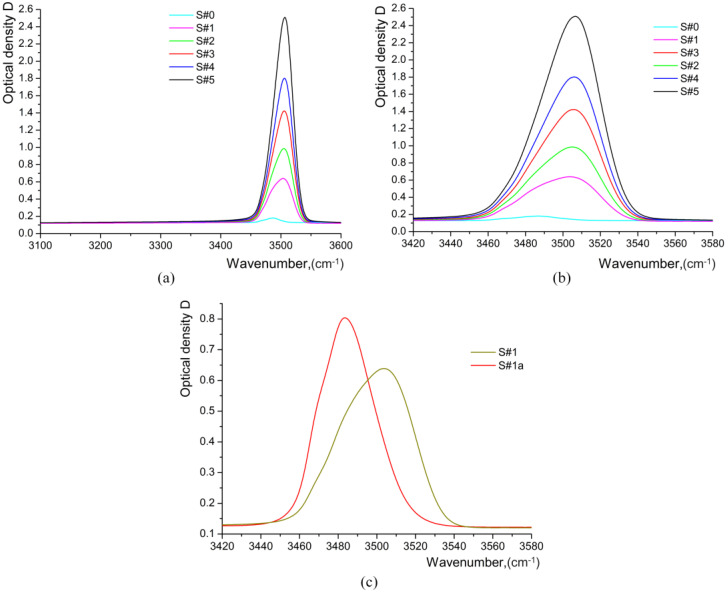
IR absorption spectra of all HiVac-VPE samples: (**a**) measured in the range of 3100–3600 cm^−1^; (**b**) measured in the range of 3420–3580 cm^−1^; (**c**) IR absorption spectrum of S#1 sample in comparison with the spectrum of as-annealed S#1a sample.

The absorbance curves in Figure 12 imply the existence of three OH bands around 3500 cm^−1^, characteristic of the presence of substitutional protons, and a very wide band with a maximum at about 3300 cm^−1^, due to the presence of interstitial protons, which are randomly distributed in the lattice.

A very weak broad band centered at 3240–3300 cm^−1^ is observed in the IR absorption spectra of all the samples. Such a small intensity (peak value of optical density *D*) of this OH-band at 3280 cm^−1^ in IR absorption spectra is clear evidence of the absence of *β_i_*-phases in the HiVac-VPE waveguides. This band has a significant intensity in IR absorption spectra of PE waveguides with the *β_i_*-phase, as reported in the literature [9,11,15].

**Figure 13:**
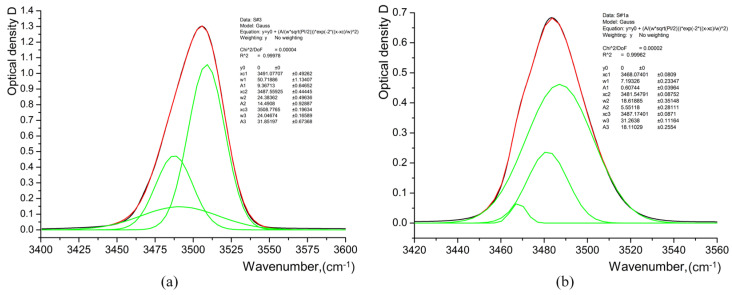
Gaussian fit and decomposition of OH-band at 3400–3500 cm^−1^ in IR absorption spectra of (**a**) S#3 sample; (**b**) S#1a sample.

The decomposition of IR absorption spectra gives the three main components for the stretching of the OH-band at about 3466–3470 cm^−1^, 3481–3488 cm^−1^, and 3508–3510 cm^−1^, as shown in Figure 13. Three bands in the region of OH stretching vibrations are indicated on the LN crystal stoichiometry (CLN in our case), and the α phase of H_x_Li_1−x_NbO_3_ is within the range of 3466–3490 cm^−1^. The bands within the range of 3496–3520 cm^−1^ indicate the presence of *κ*_1_, *κ*_2_, and *β*_i_ phases of H_x_Li_1−x_NbO_3_ [6,10,11,19,21].

Analysis of 1st and 2nd derivatives confirms the presence of such components of the OH-stretching band and demonstrates the existence of the extra component at 3496–3498 cm^−1^. The high-frequency component (3508–3510 cm^−1^) at the decomposition of the OH-band may be related to the highly protonated *κ*_2_-phase, as the *β_i_*-phases have the specific component at a higher frequency ≥ 3512 cm^−1^ as reported in the literature [9,12,15,18]. It is important to note that integral intensity *I* (*I =* ∫*D*(ν)dν) and *D*_max_ of the OH-band demonstrate a non-monotonous dependence on √*t*. After annealing for 4 h, the bands associated with the *κ*_2_-phase are not observed in the spectra of the S#1a sample depicted in Figure 12 and Figure 13, respectively. Note that the IR absorption spectra of the annealed S#1a waveguide are typical for *α*-phase only [9,13,14,15].

### 3.4. UV-VIS Absorption

The proton exchange leads to a decrease in spontaneous polarization *P*_0_ and the magnitude of such a decrease Δ*P*_0_ is specific for different H_x_Li_1−x_NbO_3_ phases [9,15,16,17,18,19,24,33,35]. A correlation has been observed between the change in spontaneous polarization *P*_0_ and the shift of the fundamental absorption edge, i.e., the change in the bandgap Δ*E*_g_ [9,36,37]. The connection between *P*_0_ and Δ*E*_g_ can be obtained by expanding *E*_g_ in a power series with respect to *P*_0_ [36] as below:

(2) $$\Delta E_{g}=\Delta P_{0}\left(aP_{0}+b^{3}P_{0}+c^{5}P_{0}\right)\approx a\Delta P_{0}P_{0}$$

where the coefficient *a* is proportional to the square of the electron–phonon coupling constant. According to the experimental data, *a* = 0.35 eV·m^4^/K^2^, i.e., even a small change in *P*_0_ can cause an appreciable shift in the fundamental absorption band edge in the spectrum of LiNbO_3_, where *P*_0_ = 0.71 K/m^2^ [35,36]. Thus, we can determine the polarization *P*_n_ = *P*_0_ − Δ*P*_0_ for any *n* phase, using data on the change of bandgap Δ*E*_g_, which is calculated directly from the observed shift of the fundamental absorption edge [33].

We need to consider that the spontaneous polarization decreases additionally as the packing density of the crystal *η* increases; *η* is defined as the ratio of the number of ions per unit volume of the studied crystal to their number per unit volume of the perovskite crystal:

(3) $$\eta =\left(\frac{\rho_{x}}{\rho_{p}}\right)\left(\frac{M_{p}}{M_{x}}\right)$$

where *ρ* is the density, *M* is the molecular weight of a formula unit, and the subscripts *x* and *p* refer to the studied crystal and the crystal of the “ideal” perovskite, respectively. For example, *η*_0_ = 1.2 for LiNbO_3_, *η* = 1.0 for BaTiO_3_, *η*_n_ = 1.24 for the *β*_3_-phase of H_x_Li_1−*x*_NbO_3_, where *x* ≈ 0.7 [16,33,35].

Thus, any change in spontaneous polarization causes a $\Delta E_{g}=\Delta P_{0}\left(aP_{0}+b^{3}P_{0}+c^{5}P_{0}\right)\approx a\Delta P_{0}P_{0}$ change in the electro-optic coefficient *r*_13_, which can be calculated from the polarization-induced change in the bandgap energy Δ*E*_g,n_:

(4) $$r_{13,n}^{\prime }=\frac{r_{13,n}^{\prime }}{r_{13,0}^{\prime }}={\left(\frac{\eta_{0}}{\eta_{n}}\right)}^{3}\left[1+\frac{\Delta E_{g,n}}{aP_{0}^{2}}\right]$$

where *r*_13,*n*_, *η*_n_, and Δ*E*_g,n_ are the values of the parameters in the studied *n*-phase; *r*_13,0_ and η_0_ are the parameters in LiNbO_3_, *r*′_13,*n*_ is the normalized value of *r*_13,*n*_ in the *n*-phase.

**Figure 14:**
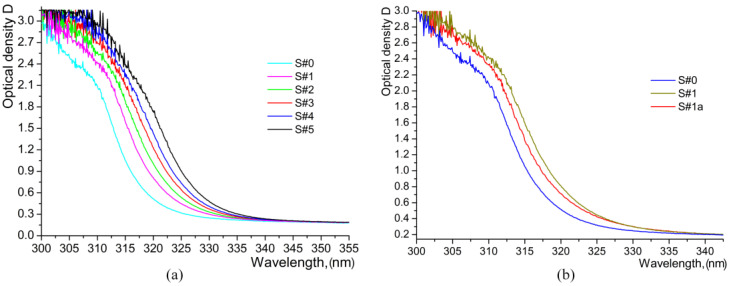
UV-VIS absorption spectra: (**a**) all HiVac-VPE exchanged samples; (**b**) S#1 sample comparative to as-annealed S#1a and virgin S#0 samples.

Comparison of the spectra of samples containing HiVac-VPE waveguides with the spectrum of the pure lithium niobate indicates a marked shift in the wavelength of the fundamental absorption band edge *λ_g_*, as depicted in Figure 14.

The absorption coefficient α was calculated from the transmittance spectra at room temperature [38] using the relation:

(5) $$T={\left(1-R\right)}^{2}\exp\left(-\alpha d\right)$$

where *T* is transmittance, *R* is reflectivity (calculated from Sellmeier’s equation), and *d* is the sample thickness. With the definition of optical density *D* = lg*T*^−1^, the following equations are obtained:

(6) $$\begin{array}{l} \alpha =\left[D+2\mathrm{lg}\left(1-R\right)\right]{\left(d\log\left(e\right)\right)}^{-1}\\ D=\alpha d\mathrm{lg}\left(e\right)-2\mathrm{lg}\left(1-R\right) \end{array}$$

For HiVac-VPE CLN samples, the UV absorption edge shifts toward a higher wavelength in comparison to virgin crystal (S#0 sample), as can be observed from Figure 14. The specific wavelengths related to the so-called “apparent edge” [38] were measured at *D* = 1.0. The specific wavelengths related to the so-called “absorption edge (AE)” [38] were determined for each sample at 20 cm^−1^ of absorption coefficient α. According to Equation (6), *D* = 1.0 corresponds to α = 37.8 cm^−1^, and α = 20 cm^−1^ corresponds to *D* = 0.62.

The direct bandgap energy *E_g_^d^* was evaluated from the plot of *α*^2^ versus *hν* (*α*^2^ versus 1/*λ*). Near the band edge, the density of state can be approximated by the parabolic band, and the absorption spectra are given by [38]:

(7) $$\alpha h\nu \propto {\left(h\nu -E_{g}^{d}\right)}^{\frac{1}{2}}$$

The abrupt rise of the absorption (*α*^2^) with incident photon energy near the band edge depicts the photon absorption due to direct allowed inter-band transition. The intercept of the straight line at the energy axis (*α* = 0) yields direct optical band gap (*E_g_*) energy.

UV-VIS absorption spectra, i.e., a band gap energy *E*_g_ evaluated from these spectra, demonstrate no marked contribution of any *β_i_*-phase. Note that *E*_g_ values for the HiVac-VPE samples, Figure 14 and Table 4 below, are lower than *E*_g_ values typical for *κ*_2_-phase in APE and SPE waveguides [9,15].

However, it significantly overcomes the values typical for the *β_i_*-phases in PE, HTPE, and SPE waveguides [9,15]. The value of bandgap energy *E*_g_ for the S#1 sample evaluated from UV absorption spectra near AE is significantly smaller than the values for the other samples. It is caused by the overlap of the absorption spectrum of the thin sublayer of *κ*_2_-phase with the spectra of the thicker sublayers of *κ*_1_ and *α* phases. The reduced values of electro-optic (EO) coefficients for HiVac-VPE waveguides are expected from UV-VIS spectra (evaluated with *E*_g_ values, Table 4). However, much smaller values are expected from the Raman spectra, as the intensity of the band at ~253 cm^−1^ is strongly attenuated within the main part of these waveguides (Figure 5). The band at ~630 cm^−1^ is also attenuated and strongly overlapped with the more intensive bands at 645–670 cm^−1^ in Raman spectra of *κ*_2_-phase (Figure 4 and Figure 5). It is established [17] that the main contributors to EO coefficients are the A_1_(TO) bands observed at ~253 cm^−1^ and ~630 cm^−1^. The nonlinear optical properties are also related to these bands. Consequently, an attenuation of their intensity is associated with the decrease of EO and NLO properties upon proton exchange. In addition, the actual effective values of EO coefficients may be significantly smaller than the values presented in Table 4 due to a structural disorder similar to APE and SPE waveguides [15,39].

According to the previous findings reported by literature [9,15,33], the UV-VIS data on the direct bandgap energy show that the annealed S#1a waveguide should contain the *κ*_1_-phase (Table 4 and Figure 14). However, IR absorption, IR reflection, and Raman spectra are typical for *α*-phase. Thus, we assume that the annealed S#1a waveguides contain the highly protonated *α*-phase, which has the anomalously high value of Δ*E*_g_. It may be regarded as an important peculiarity of the HiVac-VPE process. It means that the post-HiVac-VPE annealing can be used to produce high-quality waveguides containing only the *α*-phase.

## 4. Conclusions

In this article, we investigate the phase composition, depth profile, and electro-optic properties of waveguides fabricated using the HiVac-VPE technique through optical spectroscopy methods such as Raman spectroscopy, IR reflection, IR absorption, and UV-VIS absorption. Each phase of H_x_Li_1−x_NbO_3_ exhibits a distinct spectrum, enabling the identification of interesting and unique information on the waveguides under study.

Raman spectra measured in all studied geometries demonstrate the layered multiphase structure of the HiVac-VPE waveguides: the *κ*_2_-phase in a top sublayer (with the anomalous thin near-surface part), the *α*-phase in a depth-thick sublayer, and some phase in a transient thin sublayer placed between the *κ*_2_-phase sublayer and *α*-phase sublayer, respectively. Through low-frequency Raman scattering (LFRS), we obtained evidence of significant structural disorder induced by proton exchange in all HiVac-VPE waveguides. The tail of the broad LFRS for all waveguides strongly increases with increasing HiVac-VPE exchange time.

The IR reflection spectra of the studied HiVac-VPE waveguides show additional bands that are absent in the spectrum of the virgin niobate crystal. The frequencies of these additional bands coincide with the characteristic frequencies of the librational vibrations of the OH groups detected by Raman spectroscopy methods in other crystals. This fact, together with the presence of a characteristic dependence of their frequencies on the proton concentration, allows us to assign all components of the additional bands to librational vibrations of OH groups at different lattice positions and ordered by means of weak hydrogen bonds of different lengths. These OH-libration bands were assigned to the κ_2_-phase and to both the *κ*_1_-phase and α-phase because the *κ*_1_-phase and α-phase have the same frequency for this band in Raman spectra, respectively. The bands attributed to *β_i_* are not observed in the IR reflection spectra of all investigated HiVac-VPE waveguides.

Based on experimental data on the shift of the fundamental absorption edge, we have quantitatively estimated the electro-optic coefficient *r*_13_. Electro-optical properties of the HiVac-VPE waveguides have been found to be dependent on the phase composition.

In addition, the post-HiVac-VPE annealing process allowed the fabrication of waveguides containing highly protonated *α*-phase only.

In our investigation, we have obtained interesting results that have not yet been reported, allowing us to gain insight into the origin of the optical features of the recently reported HiVac-VPE waveguides compared to those manufactured through already known and extensively used proton exchange techniques within the scientific community.

Given the peculiarity of HiVac-VPE waveguides, they hold the potential for fabricating photonic nonlinear devices and electro-optic modulators based on high-index contrast waveguides in the LiNbO_3_ platform.

## Figures and Tables

**Table 1:** Index contrast ∆*n_e_* at *λ* = 632.8 nm of planar waveguides fabricated by the HiVac-VPE process.

Sample	Exchange Duration *t*(h)	Δ*n_e_*
S#1	1 h	0.1014
S#2	2 h	0.1027
S#3	3 h	0.1031
S#4	4 h	0.1033
S#5	5 h	0.1034

**Table 2:** References on Raman spectra parameters vs. H_x_Li_1−x_NbO_3_ phase. I_690_ and I_630_ are intensities measured at 630 and 690 cm^−1^ in raw Raman spectra without decomposition of the band on elementary components in accordance with reference [13].

Phase	I_690_/I_630_ [13]	I_690_/(I_630_ + I_690_) [13]	I_690_/(I_630_ + I_690_) [9]	*ν*_x_(cm^−1^) [9]
*κ*_1_	0.43 ÷ 0.58	0.31 ÷ 0.37	0.19 ÷ 0.30	645 ÷ 654
*κ*_2_	0.72 ÷ 0.79	0.42 ÷ 0.46	0.34 ÷ 0.46	657 ÷ 669
*β*_1_	0.96 ÷ 1.10	0.48 ÷ 0.52	0.48 ÷ 0.52	687.5
*β*_2_	1.23	0.55	0.55	687.5
*β*_3_ and *β*_4_	1.64	0.62	0.62	690

**Table 3:** Depth for sublayer boundaries for different phases. The duration of the HiVac-VPE process is denoted by *t*.

Sample	*t* (h)	*d*_k2_/*d*_k1_/*d*_α_ (μm) (m-Lines Data [1])	Plateau in Plot I_690_/(I_630_ + I_690_) vs. *t*	*d_k_*_2_ (μm)	*d_k_*_1_ (μm)	*d_α_* (μm)
S#1	1	0.62/1.1/3.3	1.3	1.6	2.3	3.3
S#1a	1 and 4 h (annealing)	-	-	-	-	≈9.0
S#2	2	1.78/2.1/4.4	1.76	2.3	2.6	4.3
S#3	3	2.30/3.5/6.6	2.39	2.6	3.0	5.0
S#4	4	2.80/4.0/6.6	2.71	3.6	4.3	6.0
S#5	5	3.30/4.6/10.9	3.29	3.6	4.3	>8.0

**Table 4:** The data on apparent edge, absorption edge (AE), bandgap energy shift Δ*E_g,n_*, and the normalized values of the electro-optic coefficient *r*_13,n_′ evaluated from Δ*E*_g,*n*_. Bandgap energy shift Δ*E_g_,_n_* = *E*_g_,*_n_* − *E*_g_,_0_ is bandgap shift induced by proton exchange in a *n*-sample; *E_g_*,_0_ is bandgap energy in a virgin CLN (S#0 sample); *E_g,n_* is bandgap energy in a *n*-th sample; *r*_13_,_n_**′** are values of the electro-optic coefficient, that were evaluated from Δ*E_g,n_*.

Sample	Apparent Edge (nm)	AE (nm)	*E_g,n_* (eV)	Δ*E_g,n_* (eV)	*r*_13,n_′
S#0	315.30	318.55	3.936	0	1
S#1a	317.20	321.05	3.908 ÷ 3.914	−(0.022 ÷ 0.028)	0.76 ÷ 0.79
S#1	318.33	322.10	3.887 ÷ 3.893	−(0.043 ÷ 0.049)	0.66 ÷ 0.69
S#2	320.04	323.80	3.873 ÷ 3.877	−(0.059 ÷ 0.063)	0.58 ÷ 0.60
S#3	321.60	325.25	3.854 ÷ 3.858	−(0.078 ÷ 0.082)	0.48 ÷ 0.50
S#4	322.67	326.37	3.838 ÷ 3.842	−(0.094 ÷ 0.098)	0.40 ÷ 0.42
S#5	324.30	327.85	3.830 ÷ 3.834	−(0.102 ÷ 0.106)	0.36 ÷ 0.38

## Data Availability

The raw data supporting the conclusions of this article will be made available by the authors on request.
